# Teachers’ Strategies for Supporting Perfectionistic Students: Assessment and Lessons Learned in Elementary and Secondary Schools

**DOI:** 10.1177/07342829261454877

**Published:** 2026-05-21

**Authors:** Danielle S. Molnar, Melissa Blackburn, Natalie Tacuri, Dawn Zinga, Paul L. Hewitt, Gordon L. Flett

**Affiliations:** 1 Department of Child and Youth Studies, 7497 Brock University, St. Catharines, ON, Canada; 2 Department of Psychology, 7497 Brock University, St. Catharines, ON, Canada; 3 Department of Integrated Studies in Education, 5620 McGill University, Montreal, QC, Canada; 4 Department of Psychology, University of British Columbia, Vancouver, BC, Canada; 5 Department of Psychology, 7991 York University, Toronto, ON, Canada

**Keywords:** classroom, education, perfectionism, personality, students, teachers, qualitative

## Abstract

Understanding and managing perfectionistic behaviours in students is a critical challenge for teachers. Consequently, this qualitative study sought to assess the strategies that elementary and secondary teachers in Ontario, Canada use to respond to perfectionism in their classrooms. Open-ended survey responses from 197 teachers (83.25% female, M_age_ = 40.21, *SD* = 9.00; 66.50% elementary teachers) and semi-structured interviews with a subsample of 26 teachers (84.62% female, M_age_ = 41.19, *SD* = 10.95; 65.38% elementary teachers) were analyzed using thematic analysis based on descriptive phenomenology. Findings indicated that teachers employ a range of strategies, such as encouraging a growth mindset, offering individualized support, fostering strong teacher-student relationships, and cultivating self-care among their students. Despite their efforts to build resilience and strengthen students’ sense of self, some teachers highlighted the persistent nature of perfectionism and expressed frustration with their limited capacity to effectively support perfectionistic students. These findings highlight the importance of recognizing perfectionism as a significant educational issue and suggest that insights from teachers’ observations and classroom strategies could inform psychoeducational assessment and targeted professional development.

## Introduction

Perfectionism (requiring perfection from the self and/or others, Hewitt & Flett, 1991) has garnered substantial attention due to its increased prevalence among young people (Curran & Hill, 2019) and its implications for well-being, relationships, and academic performance (Flett & Hewitt, 2022). Young people exhibiting perfectionistic tendencies experience intense pressure to meet excessively high standards, fear of failure, self-criticism, and tend to remain committed to perfectionism despite its immense consequences (Molnar et al., 2023). Over the past decade, advances in the assessment of perfectionism have underscored the importance of multidimensional measurement, construct clarity, and refinement of assessment tools (e.g., Flett & Hewitt, 2016, 2020). More recently, calls have been made to move beyond static trait measurement toward more person-focused and contextually grounded approaches that capture how the need to be perfect is experienced and expressed in everyday settings (see Flett & Hewitt, 2024).

Despite these advances in measurement and theory, comparatively less attention has been devoted to how perfectionistic tendencies are recognized and addressed within applied educational contexts. Although research has extensively examined associations between perfectionism and various outcomes among students (Flett & Hewitt, 2022), far less is known about how teachers interpret and respond to perfectionistic behaviours in their classrooms (Rice et al., 2016). Most research has either focused on student self-reports (e.g., Fletcher & Neumeister, 2017) or on perfectionism among educators themselves (e.g., Shim et al., 2020), with limited emphasis on the practical strategies teachers use to manage perfectionism in their students’ day-to-day experiences.

From a school-based perspective, this gap is particularly salient. Teachers routinely observe students’ behavioural, emotional, and cognitive responses to academic demands and evaluative situations, placing them in a unique position to identify observable indicators of perfectionism. These indicators may include avoidance of challenges, excessive concern over grades, constantly redoing assignments, difficulty enjoying or accepting success, strong emotional reactions to mistakes, and reluctance to submit work due to fear of failure (Molnar et al., 2023, 2026; Rice et al., 2016). Such behaviours reflect core dimensions articulated in multidimensional models of perfectionism and provide ecologically grounded indicators that may complement self-report measures. By examining teachers’ perspectives on practical strategies for working with perfectionistic students, the present study responds to previous calls for more contextually grounded and person-focused approaches to understanding the need to be perfect (Flett & Hewitt, 2014), extending prior advances in the perfectionism field into the realities of the classroom. Indeed, given their close, ongoing interactions with students, teachers may play a key role in addressing these behaviours as they arise. However, limited research has systematically examined how teachers conceptualize and implement strategies to support perfectionistic students across developmental levels, which may restrict their ability to provide effective support and place both students and teachers at increased risk for burnout and emotional distress. Accordingly, the present study investigated elementary and secondary teachers’ practical approaches to managing perfectionism in everyday classroom contexts.

### Perfectionism

According to the Comprehensive Model of Perfectionistic Behaviour (Hewitt et al., 2017), perfectionism is a multifaceted construct that operates at the trait, intrapersonal, and interpersonal levels. At the dispositional level, trait perfectionism is a deeply ingrained need for flawlessness, either for oneself or for others, which drives persistent thoughts and behaviours aimed at meeting these perfectionistic standards. Different models of trait perfectionism have been put forward. As outlined by Hewitt and Flett (1991), the three core trait dimensions are self-oriented perfectionism (SOP), other-oriented perfectionism (OOP), and socially prescribed perfectionism (SPP). SOP refers to an individual’s internal drive to set excessively high standards for themselves and strive relentlessly for flawlessness. OOP involves unrealistic expectations for others, often leading to censure when others do not meet these standards. SPP is the belief that others expect perfection and will reject or criticize the self if these expectations are not met (Hewitt & Flett, 1991). Alternatively, Frost et al. (1990)’s model underscores six dimensions of trait perfectionism: personal standards, concern over mistakes, doubts about actions, parental expectations, parental criticism, and organization. A more recent framework utilized the Big Three Perfectionism Scale (Smith et al., 2016) to assess the three broad traits of rigid perfectionism, self-critical perfectionism, and narcissistic perfectionism.

Beyond the trait level, the intrapersonal component of perfectionism is known as perfectionistic cognitions and is characterized by an incessant and self-critical internal dialogue fixated on the need to be perfect (Flett et al., 2016, 2018; Hewitt et al., 2017). Perfectionistic cognitions include a variety of automatic thoughts and ruminations occurring both consciously and unconsciously, such as anticipatory thinking, rumination on perceived mistakes and failures, and self-critical thoughts. Finally, the interpersonal level of perfectionism emphasizes the behavioural expression of perfection via deliberate efforts aimed at showcasing a perfect image, driven by three primary strategies (Hewitt et al., 2003, 2017). Perfectionistic self-promotion is a strategy focused on actively presenting perfection via outward displays. Additionally, perfectionistic self-presentation includes two concealing strategies: non-display of imperfections and non-disclosure of imperfections. While both aim to avoid revealing flaws, the former involves concealing behaviours that might be perceived as imperfect, whereas the latter refers to the avoidance of sharing personal information that could be negatively evaluated (Hewitt et al., 2003, 2017). In sum, perfectionism can manifest in various trait, intrapersonal, and interpersonal ways, likely making it challenging for teachers to effectively support perfectionistic students.

### Perfectionism in the Classroom

Research underscores the relevance of perfectionism in school settings (e.g., Fletcher & Neumeister, 2017). A growing body of work supports links between trait perfectionism and academic achievement, illustrating how different dimensions of perfectionism may hinder or enhance academic performance (see Flett & Hewitt, 2022). A notable meta-analysis, for example, highlights how SPP, doubts about actions, concern over mistakes, and discrepancy are linked to underachievement, whereas SOP, personal standards, and high standards are associated with some gains in academic performance (Madigan, 2019). Moreover, Madigan (2019) found that these relations were consistent across primary, secondary, and tertiary levels of education. Further, research emphasizes the complexity of these relations, revealing that academic performance may also predict increases in perfectionism over time (e.g., Damian et al., 2016; Negru-Subtirica et al., 2023). For example, Damian and colleagues (2016) found that higher levels of academic achievement predicted higher levels of perfectionistic standards (including a measure of SOP) and higher levels of perfectionistic concerns (including a measure of SPP) across time among a sample of students enrolled in grades six through twelve. Notably, these findings were consistent across age groups and after accounting for feelings of academic efficacy (Damian et al., 2016).

It is important to recognize that perfectionism is linked to maladaptive outcomes beyond academic performance among young people. With respect to the educational setting, meta-analytic research has highlighted differential associations between trait dimensions of perfectionism and both positive and negative learning outcomes across elementary, secondary, and tertiary levels of education (Osenk et al., 2020). Specifically, discrepancy, concern over mistakes, doubts about actions, and SPP were linked with maladaptive learning outcomes (e.g., procrastination, burnout, stress, test anxiety). Conversely, SOP, high personal standards, and high standards were related to outcomes that support academic success (e.g., academic self-efficacy, engagement, academic satisfaction; Osenk et al., 2020). More broadly, trait perfectionism has been consistently linked to a variety of adverse well-being outcomes among children and adolescents, including poorer mental health. For instance, a systematic review and meta-analysis by Lunn et al. (2023) found that both perfectionistic strivings (e.g., SOP) and perfectionistic concerns (e.g., SPP) are associated with various forms of psychopathology among young people, including anxiety and obsessive-compulsive symptoms, whereas perfectionistic concerns were also associated with depressive symptoms. Collectively, these findings highlight how perfectionism, particularly when rooted in external pressures and self-critical tendencies, can undermine both mental health and academic well-being. This makes it critical for teachers to be able to address perfectionistic behaviours, as students may struggle not only academically but also emotionally.

### Managing Perfectionism in the Classroom

Given that perfectionism may contribute to unfavourable learning outcomes, it is unsurprising that perfectionism and fear of making mistakes have been identified globally by teachers as among the top problems experienced by students (Rescorla et al., 2007). Yet, there remains a paucity in research regarding how teachers address these perfectionistic tendencies within their instructional practices. While research is limited, there are some key findings that offer valuable insights into teacher strategies that may help mitigate student perfectionism. First, Flett and Hewitt (2014) recommend that educators focus on cultivating resilience in students by encouraging them to view challenges as opportunities for growth rather than threats to self-worth. Further, they emphasize the role of self-compassion to help students develop a more balanced perspective on their achievements and failures, reducing the intense fear of making mistakes and allowing students to focus on the learning process rather than on the pursuit of unattainable perfection.

Moreover, targeted interventions designed for perfectionistic students have shown promise. For instance, Zousel et al. (2013) found that using cartoons and bibliotherapy to teach elementary school students (i.e., grades one through three) about perfectionism helped foster healthier perspectives on mistakes and self-acceptance. Similarly, Mofield and Chakraborti-Ghosh (2010) found that gifted middle school students with elevated perfectionistic tendencies demonstrated a decrease in concern over mistakes and high personal standards following an affective curriculum that focused on developing the self-awareness and skills to cope with high levels of pressure, the expectations of other people, and the unhealthy sides of perfectionism through reflective activities, role-playing, bibliotherapy, and relaxation techniques.

Research also emphasizes the role of teacher-student relationships in mitigating the harmful effects of perfectionism. For instance, Fredrick et al. (2017) demonstrated that supportive teacher relationships among students in grades six to eight buffered the adverse effects of perfectionism on poorer mental health by providing a safe space for students to take academic risks and learn from mistakes. Similarly, in their short-term longitudinal design, Domocus and Damian (2018) found that adolescents (i.e., grades nine through twelve) who perceived greater teacher support experienced lower levels of SOP and perfectionistic concerns over time. Collectively, findings highlight the importance of positive student-teacher relationships in supporting perfectionistic students.

Finally, Schruder et al. (2014) outlined effective strategies teachers can use to support perfectionistic students. Importantly, these researchers emphasized that there is no one-size-fits-all approach to intervening with perfectionism, as each student’s behaviours and needs are unique. However, they offered a range of approaches that can assist teachers in addressing perfectionism, including fostering a supportive learning environment, normalizing mistakes, helping students reflect on their emotions, and setting realistic goals. Further, they recommended that techniques such as tracking progress and practicing stress-management activities can help students cope with perfectionistic tendencies (Schruder et al., 2014).

Unfortunately, few studies have examined teacher perspectives on student perfectionism. Indeed, most research has focused on student perceptions or relied on theoretical frameworks to guide recommendations, without examining how teachers assess and implement these strategies. One notable exception is a qualitative study in which 98 elementary school teachers completed in-depth interviews responding to vignettes describing problem students (Brophy & Rohrkemper, 1989). Two vignettes described perfectionistic students. Findings showed that teachers had a solid understanding of student perfectionism, with many already implementing strategies to address it, including efforts to shift perfectionistic mindsets, provide encouragement, and teach as well as model effective coping strategies. More recently, a qualitative study examined teachers’ perspectives of perfectionism in classroom settings, highlighting their understanding of its impacts on student learning and well-being (Molnar et al., 2026). However, unlike Brophy and Rohrkemper’s (1989) work, this study did not directly consider the strategies that teachers use to manage perfectionism among students.

### The Current Study

The present study examined how elementary and secondary school teachers support students with perfectionistic tendencies in everyday classroom practice, addressing the need for in-depth, teacher-centred accounts of how such strategies are enacted in real educational settings. Assessments were conducted via open-response survey questions and semi-structured interviews with teachers located in Ontario, Canada. We then used thematic analysis based on descriptive phenomenology (e.g., Sundler et al., 2019). Analyses focused on understanding teachers’ perspectives on practical strategies for working with perfectionistic students.

## Methods

### Data Collection

Data for this study was drawn from a multimethod longitudinal project examining teachers’ perspectives, experiences, and well-being during the COVID-19 pandemic in Ontario, Canada. Data was collected at three time points between July 2021 and April 2022. Participants were recruited via social media advertisements and online news articles. Eligibility criteria included: (1) certification with the Ontario College of Teachers, and (2) a minimum of 2 years of teaching experience in Ontario. Eligible participants were invited to complete three online surveys approximately 2 months apart. Each survey consisted of demographic questions, validated scales, and open-response questions. The surveys were completed on participants’ personal devices via Qualtrics. Additionally, a subset of teachers was invited to complete a 60–90 minute semi-structured interview at each time point. Interviews were conducted online via video conferencing and were audio and video recorded for transcription. We used a purposive sampling approach to ensure an approximate balance of participants based on self-identified perfectionist status and their roles as either elementary or secondary school teachers. Participants were compensated with virtual gift cards for each survey and/or interview completed at each time point. The university ethics board approved all procedures for this study.

The data for the present study were drawn from the open-response questions from the baseline survey (completed July to November 2021) as well as from the baseline interviews (completed July to October 2021; see Supplemental Material A for interview guide). While both the surveys and interviews broadly examined teachers’ experiences with perfectionism in education and the impact of the pandemic on teaching and learning, this study focused on the strategies used by teachers when working with perfectionistic students.

### Participants

The broader sample of survey participants included 197 teachers between the ages of 25 to 69 years (*M* = 40.21, *SD* = 9.00). Most participants identified as women (*n* = 164; 83.25%), with 16.75% of participants identifying as men (*n* = 33). The sample was predominately White (*n* = 178; 90.82%). The remainder identified as Asian (*n* = 4; 2.04%), Indigenous (*n* = 3; 1.53%), Latin (*n* = 2; 1.02%), Black (*n* = 1; 0.51%), or Other (*n* = 8; 4.08%). One participant did not indicate their race. Teachers reported the following annual family income ranges: $0 to $49,999 (*n* = 10; 5.08%), $50,000 to $99,999 per year (*n* = 42; 21.32%), $100,000 to $149,999 (*n* = 42; 21.32%), and $150,000 to $200,000 or more (*n* = 89; 45.18%). Fourteen participants did not respond to this item (7.10%). Teachers also reported their self-identified perfectionist status, with 103 teachers (52.28%) affirming perfectionistic identification, and 92 teachers (46.70%) not identifying as perfectionists. Two teachers (1.02%) did not respond to this item.

Most teachers (*n* = 169; 85.79%) held full-time teaching contracts, while four teachers (2.03%) had part-time teaching contracts, seventeen teachers (7.63%) were on limited term contracts, and six teachers (3.05%) worked as occasional or supply teachers. One teacher (0.50%) did not report their position. On average, the teachers had 13.96 years of teaching experience (*SD* = 8.15; range = 2–48 years). Two-thirds of participants were elementary school teachers who taught grades kindergarten through eight (*n* = 131; 66.50%) and approximately one-third were secondary school teachers (*n* = 66; 33.50%) who taught grades nine through 12. Many teachers also reported facilitating extracurricular activities (*n* = 128; 65.98%).

The subset of participants who completed an interview at the first time point included 26 teachers between 25 and 69 years old (*M* = 41.19, *SD* = 10.95). This sample was mostly women (*n* = 22; 84.62%), with four participants identifying as men (15.38%). Most participants were White (*n* = 24; 92.31%). Teachers reported the following ranges for annual family income: $50,000 to $99,999 (*n* = 6; 23.08%), $100,000 to $149,999 (*n* = 8; 30.77%), and $150,000 to $200,000 or more (*n* = 10; 38.46%). Two teachers (7.69%) did not report their annual family income. Eleven (42.31%) identified themselves as perfectionists, while 15 (57.69%) did not identify as perfectionistic.

Most teachers who completed an interview held permanent, full-time contracts (*n* = 23; 88.46%), one participant (3.85%) held a permanent, part-time contract, and one participant (3.85%) held a limited term contract. One participant did not report their position. Teachers reported having teaching experience ranging from 2 to 48 years, with an average of 15.85 years (*SD* = 10.77). Approximately two-thirds of the teachers were elementary school teachers (*n* = 17; 65.38%) while approximately one-third were secondary school teachers (*n* = 9; 34.62%). Seventeen teachers reported acting as a teacher or coach for extracurricular activities (65.39%).

### Qualitative Credibility Efforts

The goals, materials, and design of the overarching project were preregistered before data collection began via the OSF registry (see https://osf.io/vj9kf/*)*. Additionally, the specific aims of the current paper were registered after data collection was completed but before the final stages of analysis took place (see https://osf.io/fk9xj/*)*.

To ensure credibility, the research team employed a range of strategies throughout the research process. Broadly, these credibility strategies were guided by the central principles of a descriptive phenomenological approach to thematic analyses (e.g., Dahlberg et al., 2008) which include maintaining an open mind to the accounts shared with the research team during the interviews by critically reflecting on researchers’ own pre-understandings (e.g., beliefs, theory, and assumptions that may restrict researcher openness) throughout analyses. More specifically, these strategies included personal responsibility, reflexivity, cross-checking, and positionality (see Supplementary Material B for more detail).

### Data Analysis

In the first stage of analysis, the interview and open-response data were analyzed separately. With respect to the interview data, transcripts were inputted into NVivo Transcription (QSR International Private Limited, 2020) and were cross-checked against the original audio files for accuracy. Corrected transcripts were imported back into NVivo to begin the coding process. We followed the principles of descriptive phenomenology (Sundler et al., 2019), consensus coding (Zinga et al., 2013) and employed an inductive-deductive thematic analysis approach (Braun & Clarke, 2021; Fereday & Muir-Cochrane, 2006) to guide the coding of the interview data.

The coding process was led by the third author, who has extensive experience in qualitative methods, alongside an undergraduate research assistant trained in qualitative analysis. The coding team was supervised by the first and fourth authors, who are experts in perfectionism research and qualitative methods, respectively (see Supplemental Material B). The coders independently reviewed a subset of transcripts and developed a list of potential inductive codes. The full coding team then discussed the initial code list and identified potential deductive codes associated with perfectionism theory. Once consensus was reached, a coding guide was created, consisting of six deductive, theory-based codes and thirteen inductive codes (see Supplemental Material C). Please note that this codebook was generated for the perfectionism section of the baseline interview more broadly and, as such, not all codes were relevant to the present analyses (see Supplemental Material D). The primary coders then coded all transcripts.

Once coding was complete, the analysis team organized responses to each interview question, as well as those corresponding to each inductive and deductive code. A similar process was employed for the first stage of analysis for the open-response questions. Specifically, the first author organized the open-response entries to the following question: “How do you deal with perfectionistic students? What strategies do you use?”. The team then met to discuss the relevant categorizations and connections across interview questions, codes, and the selected open-response question that reflected broader themes relating to teachers’ strategies for working with perfectionistic students. The analysis team collaborated in constructing a thematic map representing these strategies. The thematic map was primarily guided by the open-response data given that the surveys were administered to the entire sample and, as such, represented a broader range of teacher experiences compared to the subset of participants who completed a baseline interview. The final thematic map was reviewed by the analysis team to ensure an accurate reflection of participants’ experiences and distinguished between responses prompted directly by interview questions or by the open-response survey question and those that emerged organically throughout the interview.

Finally, the proposed themes were analyzed by the second and third authors to check for potential group differences based on perfectionist status, gender, teaching level (i.e., elementary or secondary) and years of experience (i.e., Novice: 1–5 years, Intermediate: 6–10 years, Experienced: 11–15 years, Veteran 1: 16–20 years, Veteran 2: 21+ years). Demographic information to determine group status was collected through the baseline surveys (see Supplemental Material E). The second and third authors were selected to complete this stage of analysis given their expertise in perfectionism research and qualitative methods, respectively. Each theme and sub-theme were re-categorized by responses per group (e.g., perfectionists versus non-perfectionists) to determine whether there were any notable differences in responses endorsed by group as well as in the content, length, and detail of responses across groups. Both the second and third authors concluded independently that there were no major group differences, such that any one group was strongly overrepresented in any given theme. However, in some cases, there were practical differences in the ways that elementary school teachers versus secondary teachers applied different strategies for working with perfectionistic students. These differences are noted in the Results section below, when applicable.

## Results

### Overview

The following five themes are organized in a way that reflects how teachers themselves spoke about their strategies in working with perfectionistic students. This organization remains true to teachers’ perspectives while also prioritizing accessibility for educators who may wish to draw on these findings in their own classrooms. It is noteworthy that while teachers appeared to be operating within Ontario, Canada’s Multi-Tiered System of Supports for School Mental Health (MTSS; School Mental Health Ontario, 2026), they did not explicitly reference this model in their open responses in the survey or interview responses. Instead, teachers focused on the practical strategies and decisions shaping their day-to-day classroom practices. The first theme, Growing Young Minds, reflects teachers’ incorporation of a growth mindset within their teaching philosophies at the classroom level to encourage flexibility among all students. The second theme, It’s About Time and Boundaries, captures classroom policies surrounding expectations, deadlines, and resubmissions, including how these policies are either reinforced or adjusted for perfectionistic students. The remaining themes (Dismantling Perfectionism, Individualized Approaches to Goal-Oriented Learning, Self-Care is Key) collectively reflect teachers’ emphasis on the need for flexible, individualized support when working with perfectionistic students, with a particular emphasis on relationship building and the importance of one-on-one conferencing. Each theme is discussed in detail in the following subsections (see Table 1 for a visual depiction of themes).

**Table 1.** Summary of Themes

Theme	Summary
Growing Young Minds	Many teachers made efforts to promote a growth mindset as part of their classroom pedagogy to address perfectionism among students This included instilling positive attitudes towards mistakes, encouraging students to take risks, and scaffolding an appreciation of the learning process over the end product (e.g., grades)
It’s About Time and Boundaries	Many teachers emphasized the importance of clear expectations for their perfectionistic students While some teachers offered perfectionistic students flexibility with respect to deadlines and allowed resubmissions, most emphasized the importance of maintaining firm boundaries with perfectionistic students to help them learn time management skills and how to accept their work as good enough
Dismantling Perfectionism	Several teachers mentioned speaking one-on-one with their perfectionistic students to directly address perfectionism in the classroom This included conversations aimed at understanding the roots of each student’s perfectionistic tendencies and at shifting away from the need to be perfect in favour of celebrating their efforts and successes Several teachers emphasized the heightened need for positivity and kindness when working with perfectionistic students by providing them with validation, reassurance, praise and encouragement
Individualized Approaches to Goal-Oriented Learning	Many teachers highlighted the importance of individualized support geared towards each student’s individual goals Teachers often discussed providing feedback on early drafts of assignments as well as reviewing feedback on graded work
Self-Care is Key	Many teachers mentioned the importance of providing perfectionistic students with coping strategies to mitigate their high levels of stress and anxiety, including mindfulness, meditation, breathing exercises, social stories, exercise, sleep, and relaxation, and taking breaks Interestingly, mental health conferencing with teachers seemed to be a valuable tool for students in and of itself

A consistent trend among the themes was the notion of flexibility. Teachers highlighted both the importance of fostering greater flexibility among perfectionistic students to counter rigid beliefs about success and failure, and the need to adopt flexible pedagogical approaches when working with young people struggling with perfectionism. Although some themes seemed to reflect contradictory practices (e.g., maintaining firm deadlines versus offering individualized accommodations), flexibility nevertheless emerged as a guiding principle across teachers’ accounts, as teachers adapted their approaches in response to students’ needs and contextual demands.

Further, it is noteworthy that a handful of teachers described feeling constrained in their ability to support perfectionistic students, citing difficulties in changing these patterns, limited time, and the pressure to meet curricular and school expectations. For instance, one teacher aptly captured how it often requires an entire school year to make moderate progress in reducing perfectionistic tendencies among her students:

It was really sweet. At the end of the year, she thanked me for helping her understand that it’s okay to make mistakes. […] And so it’s just really changing that mindset. But it takes the whole year to do that. It really does. And even after the year, it’s not like they’re not perfectionists anymore. It’s just that they understand that that’s how they are and how to cope with that and deal with it to bring some of that anxiety down.

Moreover, some teachers described how they would like to shift focus away from grades and towards soft learning skills, but were somewhat restricted by requirements from their school administrations and boards (“I don’t want to provide them a marked update until we’ve gone through an entire unit […] And what I’m really looking for – obviously the grades are part of my job and I have to provide grades – but if it’s asking what’s most important to me, it’s definitely the learning skills”).

Finally, while the five key themes described in detail below captured the majority of teachers’ responses, a handful of less commonly endorsed strategies that did not cluster into these broader patterns were mentioned as well. Although infrequently reported, these approaches are noted here to reflect the full range of teacher perspectives. Specifically, some teachers reported the use of humour to lighten the experience of mistakes (“We have a class culture of laughing at our own mistakes when they are silly”), fostering independence among perfectionistic students (“I do think they look for affirmations, but I want them to answer their own questions, so they realize they’ve got it all in them already”), coordination with parents and support staff (“Parental contact. If necessary, referral to school guidance/team/emotional support staff”) and advocating for system-level changes to reduce the pressure to be perfect among students (“There is no room for mistakes, there is no room for learning. […] And that’s where I think we have such a rigid system. And there needs to be room, there needs to be space to ebb and flow”).

### Growing Young Minds

Many teachers mentioned the promotion of a growth mindset as a strategy to address perfectionism in their classrooms (“We focus a lot on growth mindset, which I think a lot of teachers are kind of adopting in their classrooms [Elementary]”), which involved developing positive and flexible attitudes towards mistakes among their students. For instance, some teachers reported that they focus on “normalizing mistakes in the classroom [Secondary]” and assuring students that “it is okay to make mistakes [Elementary].” Additionally, many teachers modelled their own mistakes to help demonstrate to students that mistakes are an expected part of the learning process. In some cases, teachers described intentionally making mistakes for the purpose of modelling (“As a perfectionist myself, I always found that showing students it’s okay to make mistakes helps the perfectionist students. I will often make mistakes on purpose to show them that it’s okay [Elementary]”) while in others, teachers explained that they would capitalize on instances where they erred within the classroom to demonstrate that mistakes are inevitable (“Acknowledge my own mistakes to model that mistakes are normal. ‘Oops, I goofed here, what can I do to fix it?’ [Secondary]”).

Beyond simply normalizing mistakes, some teachers emphasized that mistakes are a valuable part of the learning process to their students (“I want you to make mistakes and fail because that’s where you’re going to learn the most out of it, actually. [Secondary]”). Indeed, teachers reported making active efforts to frame mistakes as a positive and welcome learning opportunity (“I work hard to develop a classroom that sets [the] expectation that you try your best and take mistakes as opportunities to learn and grow. My students would tell anyone that came into our classroom that mistakes are when your brain grows. [Elementary]”). Further, teachers described encouraging students to step outside of their comfort zones and take risks within the educational context as part of developing a growth mindset:

It’s ok to take risks, sometimes it works out, sometimes it doesn’t and we learn from those times. I try to model that in my lessons. I’ll try a new program on the computer with my students or a new science experiment for example. Sometimes it may work, sometimes it may not. It’s important for kids to see that it’s ok to not always succeed on the first try and it’s what we take away and learn from it that’s important. [Elementary]

To help scaffold risk taking among their students, teachers designed activities and assignments that would elicit mistakes and creative solutions at both the elementary and secondary levels. For instance, one elementary school teacher described using play and games to foster risk taking among their students: “In a mostly play-based program, I provide fun opportunities to fail or make mistakes. Playing games, building structures, making predictions, etc. will often provide opportunities for students to experience adversities in a positive context.” In a parallel fashion, one secondary school teacher reported that she purposefully designs activities and lessons in the first week of each term to elicit and celebrate mistakes among the students in her drama and English classes:

It’s designed for you to A) push your boundaries, like your own personal boundaries, and B) to make mistakes, because we celebrate that in my drama class so that we understand that we are going to learn the most about ourselves as actors and as human beings through our mistakes and failures. […] It was harder in an English class, but I do my best to set my students up to make mistakes. And not out of anything malicious, but out of a desire to teach them that no author published their very first book and it’s an immediate success.

Finally, to promote a growth mindset among their students, many teachers highlighted the importance of emphasizing the value of the learning process and celebrating improvement and progress, rather than focusing on the end product:

Tell them that we strive for progress, not perfection. The Mona Lisa wasn’t painted [in one] day. […] We look at their work progressively throughout the semester and ask them to reflect on their work last year vs this year to see progress and not perfection. [Secondary]

Encouraging students to engage in learning for the purpose of learning rather than to get Level 4s or A+ grades. This includes having discussions about grades not being so important and crucial. [Elementary]

Teachers emphasized learning as a process by often discussing the importance of feedback with students (“We need to encourage kids to listen to the words and take the feedback that will help them grow [Elementary]”) and making attempts to shift their students’ mindsets away from overvaluing grades and towards focusing on learning and improvement (“A 90 isn’t the ultimate goal, it’s what did I learn? Did I improve? That’s the thinking I try to encourage [Secondary]”). Further, some elementary school teachers described exploring alternative strategies for providing feedback that de-centres lettered and numbered grades:

When they’re doing assignments, I’m using more three-point rubrics where it’s like they have met the expectations, they haven’t met it, or they’ve exceeded it. And not trying to place a number or a letter on it, but rather have they met the expectations and then what can they do to improve that? What did they do well? And they go from there. [Elementary]

Notably, although secondary school teachers did not mention the use of alternative rubrics, one secondary school teacher emulated this sentiment by withholding mark updates from his students until later in the term:

I think the thing with grades – and I’ve told my students this – is I don’t want to provide them with a mark update until we’ve gone through an entire unit because I don’t want students doing the whole 91-92-91-92 wave or getting down on themselves when they drop that one percent. […] Obviously, the grades [are] part of my job and I have to provide grades. But if it’s asking what’s most important to me, it’s definitely the learning skills this year and teaching them how to be better students and better learners. [Secondary]

### It’s About Time and Boundaries

Beyond cognitive strategies such as promoting a growth mindset, teachers also used approaches that targeted students’ behaviour more directly, particularly through classroom policies related to expectations, deadlines, and resubmissions when working with perfectionistic students. Many teachers mentioned that setting clear expectations at the class level seemed to be particularly important to their perfectionistic students (“Set co-constructed criteria with [the] class so expectations are clear [Elementary]”; “I try to be as clear as possible with instructions and expectations [Secondary”). Often, teachers indicated that perfectionistic students required additional clarification surrounding these expectations. In some cases, teachers indicated they would extend their initial discussion of assignment expectations to include specific examples of what would be required to achieve the high grades often sought after by perfectionistic students:

I have made sure over the years that […] my expectations are clear and I tell them what it means to meet expectations right from the curriculum. We go over the rubric and I say meeting expectation is a 75. However – and that’s good enough for a lot of students. […] For [the perfectionists], they want to know ‘What do I need to do to get this – you know – to get this mark? What are you specifically looking for?’ So I give them that information. I say ‘Here’s what you need to meet the expectation. Here’s what you need to do to exceed the expectation and get your 85s and your 90s.’ [Secondary]

In others, teachers reported that they often reviewed the established expectations with perfectionistic students to reassure them that they had met the expectations for their desired grade and could submit their work without further adjustments:

Perfectionist students need to be reassured that, at a certain point, they have met the requirements for the grade they want to achieve. This can be accomplished by going over rubrics, checklists, or whatever assessment tool you are using. By going over it with them, you can show them [they] have done what was asked to achieve a level 4. [Elementary]

With respect to classroom policies surrounding deadlines, there was some variation with respect to teachers’ approaches to working with perfectionistic students. In some cases, teachers reported that they allowed for flexibility with deadlines for their perfectionistic students (“Extra time and the opportunity to work until they are happy with their work [Elementary]”; “Extended timeframes for those students to achieve their individual goals [Secondary]”). However, more commonly, teachers emphasized the importance of remaining firm with deadlines for perfectionistic students, rather than permitting extensions for them to “perfect” their work (“Giving limited time or they would take too much time as they are constantly changing or adding to assignments [Elementary]”; “I find that some perfectionistic students take too long working on a task and they need to focus on completing something within the suggested timeframe even if it is not perfect [Secondary]”).

Interestingly, elementary school teachers often made use of physical timers and firm time limits within the classroom setting to help emphasize these boundaries (“Will give deadlines, within reason – ‘You need to finish up your explanation by the end of the period.’ [Elementary”). Indeed, one elementary school teacher aptly underscored how objective time limits were supportive to younger perfectionistic students, even though they would choose to continue working if they had the choice:

… the ones who cannot stop because they need to color the entire picture, that’s an instance where we work with timers. […] You know, the old-fashioned egg timers with the sand dripping down. And I’ll say ‘This is the amount of time that you have left’ and so they can see it go. And then the picture will go over on the ‘work to be finished’ table. So they can go back to it later but they have to understand that we need to move on to the next task. […] They’re just looking for someone to put limits in place. And then as soon as they know ‘Oh, finally someone is saying these are my parameters’, it becomes a lot easier for them. [Elementary]

In contrast, secondary school teachers more commonly indicated that they would assist students in developing strategies to independently manage their time and workload within the established deadlines (“Ask yourself: ‘Is it important to go above and beyond or can I do what is expected for the work and call it a day?’ Secondly, students can create a to-do list and once the tasks are done, no more revisiting the work/project. Thirdly, chunk out the work and put a time limit. [Secondary]”). Endorsing the strategy of scaffolding time management skills among perfectionistic students, one high school teacher noted that she tries “to show them that even if there’s still more work you’d *like* to do, finding ways to manage expectations and timelines is one of the most important strategies for a gifted person to practice [Secondary].”

Finally, several secondary school teachers highlighted that they adopted a flexible resubmission policy in their classrooms to support the learning process among all their students, with particular benefits for their perfectionistic students:

One thing I do for my students is that they can resubmit something after it’s been marked, if they want to go back and fix the problems I specified. This allows perfectionist students the chance to upgrade their work by fixing any mistakes which may cause them stress from a lower grade. [Secondary]

However, one high school teacher noted that he would set boundaries surrounding his resubmission policy in cases where he recognized that students were hyperfocused on achieving a perfect grade, rather than engaging with his feedback for the purpose of learning:

I do reach a point where I don’t allow them to resubmit anymore because you’re not striving for perfection. That’s not what you’re doing in this class. Sorry, but maybe in another class you can do that. Not in any of mine, you won’t ever be striving for perfection. [Secondary]

### Dismantling Perfectionism

Beyond their broader classroom philosophies and policies, teachers described several approaches to working one-on-one with perfectionistic students. First, several teachers indicated that they make attempts to directly address their students’ perfectionistic tendencies through individual conferencing (“So we try to do the one-on-one conference and we talk it out like, why are you so worried that you receive an 80 and not an 85? What’s the stress behind that? [Secondary]”). Some teachers also emphasized the importance of addressing the roots of student perfectionism on a case-by-case basis to best tailor their support for that individual student:

I think it’s specific to each kid, like every situation is different, the way they show it is different. […] I think it’s really getting to the heart of why they are the way that they are, you know? Is it – the ones that want to impress their parents, it’s kind of understanding and talking to the parents as well to get a sense of what their expectations are and then working with it. [Elementary]

Similarly, a handful of teachers seemed to identify that perfectionistic tendencies may stem from an association between performance and self-worth and made attempts to dismantle this belief among their perfectionistic students:

I’ll look at them and say ‘Tomorrow, will you be the same person?’ ‘Yes.’ ‘If you bomb this and you get a zero on it, will you still be a good person? Will you still be a person of worth?’ Sometimes they hesitate, but most of the time they’re like ‘Yes’. And I said, ‘This one test doesn’t make or break who you are and you are going to become.’ [Secondary]

The teachers who recognized this tended to encourage their perfectionistic students to reflect on the perceived “consequences” of being imperfect, to help them realize that the costs of falling short of their high standards are not as drastic as they may believe (“Just having them create a list of ‘if this doesn’t happen, what are the consequences?’ And when they look at the consequences and realize it’s not as serious as they’re thinking, it helps with their anxiety [Elementary]”).

Beyond addressing the roots of perfectionism, these conversations often involved attempts to shift perfectionistic students’ goals away from the need to be perfect and towards celebrating trying their best, underscored by the notion that achieving perfection is not feasible (“I tell them all the things that I should be listening to myself – that it is okay not to be perfect. No one is. Trying your best is all that matters. [Elementary]”; “And I said ‘We all show up doing the best we can. And sometimes your best isn’t great compared to other people. But if it’s your best, then it’s your best.’ [Secondary]”). To emphasize this message, some teachers prompted their students to consider contextual factors that may affect their grades and performance, including the subject area, type of assignment, and shifting levels of difficulty throughout the school year (“One student wanted to know why their term two report card mark went from an A+ to an A and was concerned their performance had worsened. I explained that it was the term two work which was more difficult, not that their performance had worsened. [Elementary]”; “I also remind them that sometimes your best work is different depending on what the assignment is and that no one is great at everything which is why they have so many different teachers. [Secondary]”). Finally, some teachers attempted to highlight the downsides of perfectionism to their students in attempt to dissuade them from striving for perfection, including negative impacts to their health, relationships, and academic performance (“I hope to show the perfectionists that worrying about everything along the way is not good for their health. [Elementary”; “I try to teach students […] that perfectionism causes more issues in the long run and that focusing on trying to be perfectionistic leads us to spend more time focusing on things that do not matter in the long run. [Secondary]”).

Finally, many teachers emphasized the heightened need for positivity and kindness when confronting students’ perfectionism (“I treat perfectionistic students with a bit more care. I will congratulate them for small accomplishments. [Elementary]”):

I try not to be too hard. I always take that approach. You know, you point out the strengths with the weaknesses. I said ‘You have an awesome idea here, it’s just the way you’re presenting it is kind of weak. So let’s really make sure our readers understand your point because then this will be the strongest part of your essay. [Secondary]

Indeed, teachers underscored the notion that perfectionistic students often require validation and reassurance, both academically and emotionally (“Try to reduce their stress level by reassuring them if they feel that they are not accomplishing to their expectations. [Secondary]”; “I reassure them that it’s okay to be emotional or feel upset about it but make it about how we cope with those emotions. [Elementary]”). Moreover, many teachers emphasized the importance of providing perfectionistic students with praise and encouragement throughout the learning process:

I use a lot of praise and encouragement because my most perfectionist students are often the most unwilling to attempt new things due to fear of making mistakes. I HYPE THEM UP especially when I see them persevering or using strategies to overcome a situation they perceive as challenging. [Elementary]

With a student yesterday, I said ‘I’m so proud of you and I hope that you’re proud of you, too. […] The fact that you show up and you try and I see that you have panic attacks, you leave and you come back and you continue trying. Not everybody would do that.’ […] And today he actually asked me for help on something and I was like ‘Yes, right.’ So I think showing them more and taking that time is well worth it. [Secondary]

Notably, within their discussion of praise, teachers often made a point to mention that they try to provide praise for progress or the development of soft skills, rather than for achieving a “perfect” outcome (“I try to praise their efforts and good work. [Elementary]”; “Praise aspects of students that are not achievement based (kindness, cooperation, discussion points. [Secondary]”).

### Individualized Approaches to Goal-Oriented Learning

Many teachers discussed the importance of providing perfectionistic students with individualized support in achieving their goals:

Constant feedback lets the kids know where they are, what we’re doing, next steps, right? ‘What are your goals? What are you doing? What can you do to be more successful in this course? Here’s some hints and tips and tricks.’ So there needs to be constant feedback. [Secondary]

Importantly, several teachers highlighted that, in providing this support, it is critical to avoid comparisons between students and to tailor their support to each students’ needs (“I tend not to point to other students [Secondary]”; “Depends on the student […] Conferencing to see what works best for them [Elementary]”). Indeed, many teachers described that they would help perfectionistic students “set reasonable goals for themselves [Secondary]” and often discussed the importance of flexibility in their approaches to supporting young perfectionists in achieving their goals:

I like to give these students more choice so that they can see that there are many ways to accomplish things to a high level of success. Being able to identify this helps students to continue to be their best but also know that there is not only one path. [Elementary]

Notably, a key strategy used by many teachers was one-on-one conferencing to review assignment feedback with perfectionistic students as well as to provide input and guidance on assignments prior to submission:

Conversation and conversation and conversation. Working through thinking, explaining perspectives on grading, and understanding big picture more than one single task. [Elementary]

I like to take the time to work one-on-one with perfectionistic students and go over their work with them to provide meaningful feedback that allows them to really understand how/why they earned the marks they did. Oftentimes we will plan to go over their work before they submit it for evaluation so they have the opportunity to revise and edit ahead of time. [Secondary]

Finally, beyond individual assignments, teachers also discussed the importance of working with perfectionistic students throughout the term to set realistic goals for their class and to provide support tracking their progress towards their goals, providing support as needed:

I try to meet with them like once a month and we’ll review their goal and be like ‘Did you reach your goal? Can you set another goal? What have you been doing right? What have you been doing wrong? Like I make them write reflections and then they talk about it with me. That’s more powerful than my written feedback. [Elementary]

You have to really understand what they’re striving for and helping them relate to the real world […] Really hearing them out and trying to explain to them what they need to do in terms of what they want to accomplish. [Secondary]

### Self-Care Is Key

Many teachers at both the elementary and secondary levels mentioned the importance of providing perfectionistic students with coping mechanisms to help mitigate their high levels of stress and anxiety. Teachers referred to a wide range of potential coping strategies and approaches to self-care that they promote for perfectionistic students, including mindfulness, meditation, breathing strategies, social stories, exercise, sleep, and relaxation. Although some teachers referred to making time for encouraging self-care in their classrooms more broadly, these strategies seemed to be particularly helpful for perfectionistic students and were often applied further via one-on-one conferences:

We do a lot of the mental health, you know, calming techniques, doing a lot of the mindfulness activities to kind of de-stress them. […] And we used to write letters back and forth and through our letters he would explain to me, you know, ‘I’m understanding this now […] I’m learning a little bit more every time and it’s okay to make the mistake.’ So he showed a lot of growth with all the mental health work that we did and the mindfulness work that we did. [Elementary]

The other thing I do when I see that I’m working with a perfectionist is really take the time to try to ease the anxiety. And I try to do that from the get go because you’re always going to have one or two in class, especially if you have a university class in the upper grades. [Secondary]

Moreover, some secondary school teachers tried to link self-care with improved academic performance to entice perfectionistic students to apply the self-care strategies suggested to them by their teachers (“I also try to remind students that making time for things like healthy eating, exercise, recreation, and relaxing are all important as well and that these can have an effect on their academic performance [Secondary]”). Interestingly, mental health conferencing with teachers appeared to be an important coping mechanism in and of itself:

I’ve had students who engage in negative self-talk when they don’t excel at something right away and oftentimes they don’t realize how bad it sounds until I ask them whether they would say the same things to their friends/teachers. Showing them how to speak kindly to themselves and how to notice the little wins as they learn has had the greatest impact. [Elementary]

In the last two days, I met with every student in my Grade 12 class and talked about their research projects. And like I said, I had that breakthrough with that student yesterday who struggles with anxiety because of [perfectionism]. So that opportunity for teachers to build those relationships with students, that feedback becomes valuable because they’re open to it. If they have a teacher they don’t like and they don’t feel cares, then they may interpret that as criticism instead of ‘this is how you can grow’. [Secondary]

A few teachers mentioned the importance of encouraging breaks from schoolwork among perfectionistic students (“The students need a lot of support to help them understand that no one is perfect and that [our] brains and bodies need a break. You need to turn it off at some point [Elementary]”). Some teachers attempted to incorporate breaks into their lessons (“I incorporate a lot of ‘brain breaks’: trivia games, riddles, group challenges, etc. [Elementary]”; “Many times I would interrupt class to stop and enjoy some conversations about our pets, favourite vehicles, or the Mars Rover Landing. [Secondary]”) whereas others described offering breaks to perfectionistic students on an individual basis:

Sometimes I’ll just sit with them and guide them if they need guidance and then maybe steer them towards something else […] especially if they start to get a little frustrated. Say ‘Okay, now let’s take a break. We’ll come back – you know, change your thoughts and we’ll come back to this a little later. [Elementary]

Go on your phone for ten minutes, if that’s going to distract you, right? Then you can come back to the task at hand. […] Go on your phone for ten minutes. Go watch YouTube and then we’re going to come back. Go take a walk, right? Distractions. [Secondary]

Finally, a handful of elementary school teachers also mentioned the importance of exposing perfectionistic students to non-academic opportunities to get involved in school as another form of “built-in” distraction (“I would also encourage them at school to kind of branch out so I would give them leadership roles and take their mind off other things that was maybe causing them anxiety [Elementary]”).

## Discussion

The present study examined the tangible strategies that elementary and secondary teachers employ in their everyday classroom practices to support students with perfectionistic tendencies. We used a rigorous qualitative approach that drew upon open-response survey data and in-depth interviews. Teachers offered rich and detailed descriptions of their approaches, reflecting their extensive experience working with perfectionistic students. From these accounts, several key themes emerged, shedding light on the diverse strategies teachers use to support students with perfectionism in their classrooms. Importantly, a common thread throughout each of the key themes was the emphasis on flexibility in supporting perfectionistic students. Specifically, many teachers described efforts to foster greater adaptability in students’ academic approaches and self-perceptions, while also highlighting the importance of tailoring their own practices to students’ individual needs. Additionally, these themes reflected some trait conceptualizations of perfectionism (e.g., rigid perfectionism, self-critical perfectionism), and, as such, the current results may be regarded as indirect support for certain core aspects of perfectionism.

Moreover, the present findings have important implications for psychoeducational assessment in practice, as teachers’ accounts highlight how their ongoing observations and interpretations of students’ behaviours can meaningfully inform formal assessment processes. Rather than reacting to isolated behaviours, teachers described continuous monitoring of students’ perfectionistic tendencies, using these observations to guide flexible instructional adjustments. In this way, teachers’ accounts reflect practical, classroom-based strategies used to support perfectionistic students and provide ecologically grounded, informal insights into students’ perfectionistic tendencies. Interestingly, these practices appear to be broadly consistent with Ontario, Canada’s MTSS for School Mental Health (School Mental Health Ontario, 2026), with a strong emphasis on universal (Tier 1) strategies, as only a few teachers described targeted or intensive supports, such as coordination with families or collaboration with school-based support staff, suggesting limited representation of higher-tier practices. The prominence of Tier 1 strategies may reflect how teachers conceptualize perfectionism as more of an academic or dispositional concern than a mental health difficulty requiring intensive intervention and may have also been influenced by the study’s focus on teachers’ own classroom practices, which likely oriented responses toward individual strategies. Further research is needed to clarify the factors underlying this pattern.

The present work is consistent with Brophy and Rohrkemper’s (1989) findings showing that teachers not only recognize perfectionism but are also proactively implementing strategies to help shift perfectionistic mindsets. Teachers in their study reported putting effort into reframing students’ thinking, moving from a focus on performance and outcomes to valuing the learning process. Similarly, a key theme identified in the present work was that teachers emphasized the importance of instilling a growth mindset to promote more flexible approaches to learning and to counter the rigid, fixed perspectives often held by perfectionistic students. According to Dweck (1999; 2019), students with a growth mindset believe that their abilities can be enhanced through effort and the use of effective strategies, leading them to interpret challenges and setbacks as opportunities for growth. Conversely, students with a fixed mindset tend to view their abilities as static, often interpreting setbacks as proof of inadequacy. This distinction may be critical for perfectionistic students, who frequently feel overwhelmed by unrealistic expectations and a fear of failure (Molnar et al., 2023). Interestingly, teachers’ promotion of growth mindsets to address perfectionism among students resonates with approaches suggested in the perfectionism literature. For example, Schruder et al. (2014) recommended strategies such as normalizing mistakes, creating supportive environments, and emphasizing progress over product. Similarly, Flett and Hewitt (2014) proposed that encouraging a growth mindset could help perfectionistic students reframe setbacks as opportunities for learning rather than viewing them as signs of failure.

However, some caution is warranted with respect to endorsing a growth mindset to address perfectionism. A meta-analysis by Burnette et al. (2023) revealed that growth mindset interventions tend to be particularly effective for students who are experiencing stress or who face challenges in achieving their goals. These findings support the idea that growth mindset interventions may be especially beneficial for perfectionistic students, who often experience immense pressure and high levels of stress. Still, Burnette et al. (2023) advised that while growth mindsets can be protective for some students, they need to be carefully targeted to the unique psychological needs of the individual. Importantly, in the context of perfectionism, if students continue to hold excessively high standards, a growth mindset intervention could inadvertently lead to overwork and increased stress. Indeed, previous work has shown that perfectionistic strivings (i.e., high standards or SOP) are positively associated with growth mindsets (e.g., Mofield & Parker Peters, 2019), suggesting that while students higher in some forms of perfectionism may adopt a growth mindset, they may use it as an excuse to overwork and maintain unrealistically high expectations. Thus, careful consideration is required when implementing this strategy with perfectionistic students as combining perfectionistic tendencies with a growth mindset may lead to increased pressure, anxiety, and a cycle of overachievement that can undermine well-being in some cases.

Parenthetically, it should be noted that many of the teachers’ responses focused naturally on how perfectionistic students typically responded and reacted to mistakes and failures. It was clear that teachers recognized that perfectionistic students have orientations toward mistakes and failures that are far from adaptive. Their emphasis on these tendencies is in keeping with the need to consider perfectionism in terms of heightened sensitivity to mistakes and failures (for a discussion, see Flett & Hewitt, 2022; Yosopov et al., 2024).

Another prominent theme centred around classroom policies concerning deadlines, expectations, and resubmissions. Building on earlier growth mindset strategies that focused on cognitive self-regulation, these approaches shifted more toward behavioural regulation in how students engage with tasks. Interestingly, teachers showed considerable variation in their strategies, particularly regarding deadlines and resubmission policies. A subset of teachers discussed adjusting deadlines and allowing multiple resubmissions as a strategy to reduce pressure on perfectionistic students, reflecting a movement towards flexible classrooms that prioritize adaptability in addressing student needs. Indeed, research highlights that implementing flexible classroom policies can foster a more supportive learning environment by reducing the pressure students experience, enabling them to focus on mastering content rather than meeting rigid deadlines (e.g., Hills & Peacock, 2022).

In contrast, many teachers adopted a more structured approach by enforcing firm deadlines and limiting resubmission opportunities. They emphasized that these policies were intended to help perfectionistic students break the cycle of continuous revisions and reduce the stress of striving for unattainable standards. While this approach may seem at odds with the benefits of flexible assessment policies, it addresses key challenges associated with them. Cowan (2024) highlighted the delicate balance between flexibility and firmness, noting that while flexible deadlines can reduce stress, they may also contribute to procrastination and anxiety. This is particularly relevant for perfectionistic students, as certain dimensions of perfectionism (e.g., SPP) are linked to increased anxiety and procrastination (e.g., Bong et al., 2014; Spadafora et al., 2022; Yosopov et al., 2024). Molnar et al. (2023) similarly found that some young perfectionists feel compelled to continually redo their work, resulting in dissatisfaction and an unrelenting drive for improvement. As such, flexible deadlines and repeated resubmissions may inadvertently reinforce perfectionistic tendencies rather than mitigate them.

These contrasting approaches highlight a broader assessment challenge: determining when flexibility reduces pressure and when it may inadvertently sustain perfectionistic overwork. Teachers in the present study appeared to engage in ongoing evaluative judgements, often adjusting classroom policies based on observed student responses to high standards, perceived mistakes, and feedback. Such day-to-day observations reflect the type of ecologically grounded, person-focused assessment that the perfectionism field increasingly calls for. This is in line with broader recommendations to move the field toward more comprehensive, person-focused approaches that integrate behavioural indicators, individual differences, and situational factors (Flett & Hewitt, 2016, 2020, 2024).

The final set of strategies employed by teachers emphasized the importance of taking an individualized approach, which is consistent with calls in the perfectionism field to recognize heterogeneity among young perfectionists (e.g., Flett & Hewitt, 2022, 2024; Mofield & Chakraborti-Ghosh, 2010; Molnar et al., 2023). Specifically, teachers emphasized the importance of adopting a relational approach to individualized support and building trust through consistent, empathetic communication to help perfectionistic students feel safe enough to take risks and confront their fear of failure. The procedures adopted accord with descriptions of teachers as caring adults who convey to students that they are individuals who matter to them. Teachers often used one-on-one conferencing with students to discuss academic challenges, emotional responses to mistakes, and realistic goal setting, while also providing personalized guidance on upcoming assignments and offering additional feedback on previously graded assignments or tests. On some occasions, these conferences also served as a space for teachers to help students identify the roots of their perfectionism and explore how these tendencies were problematic in their academic and personal lives.

Importantly, while teachers did not explicitly refer to perfectionistic reactivity (i.e., the intense emotional responses to mistakes, perceived failure, or falling short; Flett & Hewitt, 2022) some of their strategies appeared to indirectly address this issue, particularly when they discussed directly confronting perfectionism among their perfectionistic students. This finding is not only consistent with results demonstrating how young people often experience heightened distress when their perfectionistic standards are unmet (Molnar et al., 2023) but demonstrate teachers’ awareness of the emotional toll associated with perfectionistic tendencies. Teachers described using one-on-one conferences to help students navigate not just high standards, but also the emotional fallout of academic setbacks. This suggests that, in practice, teachers were not only responding to the need for students to adjust unrealistic expectations, but were also, and perhaps unknowingly, helping them manage the emotional distress that often arose when those expectations were not met. Recognizing both the exacting standards and the reactivity they provoke is a vital distinction for teachers because it underscores the need to address not just what perfectionistic students strive for, but how they emotionally respond when they perceive that they have fallen short.

Beyond one-on-one conferences, teachers also encouraged students to take breaks and acknowledge their limits, providing strategies for coping with the stress of perfectionistic tendencies. One common strategy was to help perfectionistic students develop self-care techniques, such as cultivating self-compassion and managing their own expectations. Notably, these strategies are consistent with recommendations put forth by several researchers in the perfectionism field. For example, for perfectionistic students, self-compassion is a promising protective factor for prevention and intervention (e.g., Ferrari et al., 2018; With et al., 2024). Flett and Hewitt (2014) emphasized the importance of fostering self-compassion as a critical aspect of helping students cope with perfectionism. They suggest that promoting self-compassion can buffer students against their proneness to harsh self-criticism, allowing them to develop a more balanced and forgiving relationship with themselves.

However, the teachers in the current study advocated approaches that went well beyond previous suggestions. Their day-to-day insights reveal not just theoretical consistency with existing prevention models (e.g., Flett & Hewitt, 2014; Schruder et al., 2014), but also a nuanced, relational, and action-oriented understanding that complements and extends prior frameworks. Their emphasis on emotional attunement, real-time feedback, and responsive adjustment of classroom practices underscores the practical expertise teachers bring to this issue. The present results illustrate why teacher-based assessments are vital and the voices of teachers warrant attention, as they offer rich and contextually grounded knowledge that can deepen and extend existing prevention approaches to address perfectionism among young people in meaningful ways. However, it is important to recognize that some perfectionistic students may require support that extends beyond what teachers are equipped to offer. Indeed, some teachers in this study underscored that perfectionistic tendencies in students are often resistant to change and that teachers are frequently constrained in their ability to effectively support perfectionistic students due to time limitations and the need to adhere to curricular and school expectations. Given that teachers’ accounts largely reflected Tier 1 supports within Ontario, Canada’s MTSS for School Mental Health (School Mental Health Ontario, 2026), and that perfectionism tends to be deeply ingrained, frequently contributes to emotional distress, and is often resistant to change, there is a clear rationale for connecting students to Tier 2 supports when needed. Teachers are therefore encouraged to collaborate with parents and additional school staff (e.g., child and youth care workers, social workers, psychologists) to ensure students receive the necessary care and interventions when necessary (Schruder et al., 2014). Acknowledging the boundaries of their role can empower teachers with a broader perspective and reinforce that they are one part of a larger network of support for students struggling with perfectionism.

Finally, we intentionally included both elementary and secondary school teachers to capture potential differences in how strategies for supporting perfectionistic students may vary across developmental stages and instructional contexts. Research on elementary versus secondary education highlights notable distinctions in student age, developmental needs, and instructional approaches with elementary classrooms typically emphasizing foundational skills, emotion regulation, and more teacher-led support, and secondary classrooms focusing on subject specialization, independent learning, and advanced organizational skills (Baines et al., 2003). Although our results revealed that the overarching types of strategies teachers described were largely consistent across both groups, there were some differences in how these strategies were applied to accommodate developmental differences. For example, elementary teachers more often described using concrete tools like egg timers to help younger students limit redoing work, whereas secondary teachers tended to focus on helping older students to develop independent time management skills appropriate for adolescents. This pattern suggests that while the core themes of teacher responses to perfectionism did not differ by school level, their implementation was tailored to fit with the cognitive, social, and instructional demands of the learners at different developmental stages.

### Strengths and Limitations

The current study has some limitations to consider when interpreting the findings. First, most participants identified as White women, which limits the generalizability of the findings. Although the sample lacked diversity, it is important to note that it was reflective of the demographic composition of teachers in Ontario, where most teachers tend to identify as White and female (Ontario College of Teachers, 2023). Additionally, while a strength of the study was its focus on gathering insights from teachers as informed outsiders regarding student perfectionism, the findings are based exclusively on teachers’ self-reported responses, which may be influenced by social desirability or a lack of awareness regarding the full impact of certain strategies. Future research could enhance our understanding of teacher practices by incorporating observational studies, allowing for a more nuanced view of how interventions unfold in the classroom and how students respond to them. This would not only validate teachers’ self-reports but also provide a more dynamic picture of how perfectionism is addressed in practice. Similarly, while teachers shared insights into the strategies they use to address perfectionism in students, the effectiveness of those strategies was not evaluated or measured. Without an assessment of how well these approaches work in practice, it remains unclear whether the strategies have a meaningful impact on reducing perfectionistic behaviours or improving student outcomes. Future research should incorporate methods to assess the effectiveness of these approaches, such as student feedback or longitudinal tracking of academic and emotional progress, to provide a clearer understanding of which strategies are most successful in mitigating student perfectionism. Finally, the data for this study were collected during the COVID-19 pandemic, which likely affected teachers’ workloads, stress levels, and classroom conditions in ways that could have influenced their responses. However, the present analysis drew exclusively on open-response and interview data in which teachers described their typical, ongoing classroom practices for supporting students with perfectionistic tendencies, rather than pandemic-specific adaptations. Future research should nonetheless acknowledge this contextual limitation by examining whether and how teachers’ approaches to supporting perfectionistic students differ in more typical versus disrupted teaching contexts.

Despite these limitations, a key strength of the current work lies in its focus on capturing the perspectives of teachers, voices that are often overlooked in perfectionism research. By foregrounding teachers’ experiences, this study draws attention to the practical, daily challenges faced by teachers when working with perfectionistic students. Although the present study did not evaluate the effectiveness of the strategies teachers described, this descriptive focus represents a necessary foundational step, as understanding what teachers are actually doing in practice, and the contextual factors shaping their decisions, is essential before effectiveness can be meaningfully assessed. The qualitative approach adopted here yielded rich, in-depth narratives that illuminated both the strategies teachers employ and the rationale underlying their use. As teachers are typically the first to identify and respond to perfectionism in the classroom, their perspectives provide critical insight into the real-world implementation of support strategies and lay the groundwork for future research aimed at developing and evaluating contextually grounded interventions.

## Conclusion

The present study examined the practical strategies used by elementary and secondary teachers when addressing students’ perfectionistic behaviours, providing a rare and valuable glimpse into the tangible strategies they use in the classroom every day. Findings indicated that teachers are not only aware of the detrimental effects perfectionism can have on students’ learning and well-being but also employ a diverse range of strategies to support students with perfectionistic tendencies including promoting a growth mindset, individualized support, and relationship building. Although teachers often endorsed differing approaches, all teachers in this study shared a deep concern for their students and recognized the need for individualized and compassionate support. At the same time, teachers reported that they often struggled with effectively supporting perfectionistic students, highlighting the intricacies of perfectionism and the need for additional professional development and resources. These results underscore the importance of documenting teachers’ current practices as a first step toward evidence-based support and suggest that future research should examine the effectiveness of these strategies to better guide psychoeducational assessment and intervention efforts.

## Supplemental Material

Supplemental Material - Teachers’ Strategies for Supporting Perfectionistic Students: Assessment and Lessons Learned in Elementary and Secondary Schools Supplemental Material for Teachers’ Strategies for Supporting Perfectionistic Students: Assessment and Lessons Learned in Elementary and Secondary Schools by Danielle S. Molnar, Melissa Blackburn, Natalie Tacuri, Dawn Zinga, Paul L. Hewitt, Gordon L. Flett in Journal of Psychoeducational Assessment
