# Supplementary material for: Teachers’ Strategies for Supporting Perfectionistic Students: Assessment and Lessons Learned in Elementary and Secondary Schools
Source: J Psychoeduc Assess. 2026 May 21;44(8):871–95. doi: 10.1177/07342829261454877 (PMC13690266; doi:10.1177/07342829261454877)
Supplement: Supplementary file 1 [file sj-pdf-1-jpa-10.1177_07342829261454877.pdf]

Supplemental Material A: Time 1 Interview Questions

G1. Why did you get into teaching?

G2. What was the most recent grade that you were teaching or are currently teaching?

Perfectionism and Education

P1. Have you had students who you would describe as perfectionistic in your classroom? If yes, can you describe what they are like?

P2. Do you think perfectionism in kids has increased since you've been teaching? If so, in what ways?

P3. What do you think the source of perfectionism is among students? (e.g. from the self, parents, others, society)

P4. How do you deal with perfectionistic students? What strategies do you use?
   P4a. If they say they don't have any switch to: What strategies would you use if you did have perfectionistic students?

P5. How do you deal with perfectionistic parents? What strategies do you use?
   P5a. If they say they don't have any switch to: What strategies would you use if you did have students with perfectionistic parents?

P6. How important do you feel that grades are?
   P6a. How important do you feel that feedback is?

P7. How important do you think grades are to your students?
   P7a. What do you find most frustrating about dealing with students and their grades?

P8. How do you think students interpret feedback from teachers?

P9. Do you think students are experiencing more pressures these days?

P10. Where do you think the pressure comes from?

P11. Would you identify yourself as a perfectionist?
   P11a. If yes, why would you describe yourself as a perfectionist?
   P11b. Would you be the same if your perfectionism got taken away?
   P11d. What do you think the opposite of perfectionism is?
   P11c. If you could take away your perfectionism, would you choose to? Why or why not?
   P11e. If no: Do you know any teachers (without naming names) who you would describe as a Perfectionist?

P12. How do you think your own perfectionism impacts how you teach?

P12a. Or if they aren't perfectionistic: How do you think a teacher's perfectionism impacts how they teach?

P13. Do you think perfectionism affects all areas of people's lives or some parts more than others? (Domain specificity)

Pandemic and Education

C1. Can you describe what teaching was like for you prior to the pandemic?

C2. Can you please describe what teaching was like during the pandemic?

C2a. What were the best aspects (if any) of teaching during the pandemic?

C2b. What were the biggest challenges of teaching during the pandemic?

C2c. How are you feeling about returning to school in September? Are you worried about anything? Are you excited about anything?

C3. What was your workload like this past year during the pandemic as compared to pre-pandemic conditions?

C4. How do you feel the education/school experience your students had during the pandemic compares to their pre-covid education?

C4a. Do you feel that this past school year prepared students for transitions (e.g., grade 8 to grade 9, grade 12 to post-secondary)?

C5. The pandemic has created additional stress for teachers and students, how have you, and how have the students dealt with the additional stress and changing conditions? Do you think there will be long-term effects? If so, please describe

C6. Please describe any supports provided to you during the pandemic? What was helpful? What was not helpful? What do you wish would have been provided?

C6a. When did you feel supported?

C6b. When did you not feel supported?

C7. Does a parent have the right to know if teachers are vaccinated?

C7a. How much information should people have access to regarding the vaccination status of others?

C7b. Do you feel that vaccines should or should not be mandated for teachers, students, and other education staff? Why or why not?

C7c. What do you think about the possibility of 'vaccine passports' (e.g., having to carry proof of vaccination in order to access certain privileges such as businesses, flights, university dorms, camps, etc.)

C7d. If it doesn't come up: How would you feel about being required to carry a vaccine passport?"

C8. If you were in charge of the provincial government and had to decide on COVID restrictions, what would you do differently or the same (e.g., who gets vaccinated when, masking, what should be closed, school closings etc.)

Ending

E1. Is there anything else about teaching that we haven't covered that you think we should know?

E2. What do you find most rewarding about teaching?

Supplemental Material B: Additional Methods Information

Interview Research Team

Please see Table S1 below for a description of the interview team for the baseline interviews of the present study. All members of the research team received interview training from the fourth author (Ph.D., Professor, Female), given her extensive expertise in qualitative research methods. There were no pre-existing relationships between participants and the interviewer who conducted their interview. Although participants had already provided virtual consent at the beginning of the study, each interview began with an oral review of key information from the consent form relevant for the interview component of the study. All interviewers followed a script for this consent review to ensure that all participants received the same information about the interviews. All participants confirmed they understood their rights as participants and were interested in participating with the interview before the interviewer proceeded with the interview questions. Participants were not given any information about the individual interviewers outside of describing the purpose of the project during the consent review. While interviews were being conducted, the primary research team (first through fourth authors as well as the interview team described below) met regularly to reflect on the interviews, review any modifications needed to the interview guide, and discuss notable excerpts from individual interviews.

Data Analysis

Member Checking

We did not employ member checking procedures for this study as the research team agreed that teachers were likely already overburdened during the COVID-19 pandemic given the quickly changing landscape of education at the time this study took place. As such, we determined that requiring member checking would be overly taxing for our participants.

However, participants were informed that they had the right to contact the research team if they wanted to change, add, or remove anything with respect to what they shared during their interviews. None of the participants contacted the research team with a request to modify their transcript.

Data Credibility and Reflexivity

We employed a variety of credibility strategies throughout each stage of the research process underlying the current work. First, we centered the notion of personal responsibility throughout our research through our commitment to conducting our projects in ethical and fair ways and to maintaining rigorous data collection, storage, and analysis protocols to ensure the integrity and confidentiality of participant data. Further, consistent with principles of consensus coding and our descriptive phenomenological approach to thematic analysis, we engaged in reflective, consensus-building meetings throughout the coding and analysis stages. These meetings ensured that we built in cross-checks among research team members that allowed for conversations about whether our conclusions were adequately representing the experiences of our participants, as they were expressed to us, as well as about the potential rivalling explanations for the conclusions drawn based on the data, when necessary.

Finally, we consciously chose the members of our core research team (Authors 1 through 4) to balance our varying positionalities in relation to the current research. First, our research team is multidisciplinary in nature. Authors 1 and 4 were both formally trained in psychology and Author 4 has spent 22 years working in a multidisciplinary department and identifies as a multidisciplinary researcher, whose work primarily cuts across psychology, education, and sociology as well as other disciplines such as kinesiology and leisure studies. Authors 2 and 3 are both currently completing multidisciplinary doctoral degrees in child and youth studies and

education, respectively. However, Author 2 has received the bulk of her research training from a psychological perspective, whereas Author 3 has focused on applying a critical, sociocultural lens to her work thus far. Further, of relevance for the current paper, the first and second authors have expertise in perfectionism theory and research, largely from a psychological perspective, as well as their own personal experiences with perfectionism, whereas Authors 3 and 4 have a more tangential familiarity with the perfectionism literature through their collaboration with the first two authors on previous projects. In an opposite pattern, Authors 3 and 4 have extensive experience with qualitative research methods whereas the first two authors are earlier in their qualitative training and have more recently begun to employ a bottom-up, qualitative approach to understand perfectionism in young people in their work. Finally, it is worth noting that Authors 1 and 4 are both postsecondary educators who taught during the pandemic, which may have helped to shape their perceptions of the experiences of the teachers in this study.

Consistent with a descriptive phenomenological approach to thematic analysis (Sundler et al., 2019), we made concerted efforts to critically reflect on each of our pre-understandings through group discussions to ensure that any pre-existing assumptions were not restricting our openness to understanding the accounts of the teachers in the current work as they were expressed to the research team during the interviews. In other words, we incorporated reflective group discussions throughout each stage of analysis to provide a platform for each member of the research team to critically question their own pre-existing assumptions and ensure that the analysis process was allowing the data to ‘speak for itself’ rather than reflecting our collective pre-understandings. The unique combination of our research team allowed for a balanced and meaningful approach to analysis, which involved many reflective discussions of our own potential biases throughout each stage of the present research. In sum, our multidisciplinary

research team carefully balanced expertise with perfectionism from a psychological perspective and expertise with qualitative research from a broader perspective that allowed for a collaborative and rigorous research process.

Table S1

Interview Team

	Author 3	RA 1	RA 2	RA 3	RA 4
Credentials	M.A.	Ph.D.	Ph.D.	Hon. B.A.	Hon. B.A.
Occupation	Research assistant	Research associate	Research associate	Research assistant	Research assistant
Gender	Female	Female	Female	Female	Female

Table S2

Coding Team

	Author 3	RA 5	Author 1	Author 4
Credentials	M.A.	High school diploma	Ph.D.	Ph.D.
Occupation	Research assistant	Research assistant	Professor	Professor
Gender	Female	Female	Female	Female

Table S3

Analysis Team

	Author 1	Author 2	Author 3	Author 4	RA 5
Credentials	Ph.D.	M.A.	M.A.	Ph.D.	High school diploma
Occupation	Professor	Research assistant	Research assistant	Professor	Research assistant
Gender	Female	Female	Female	Female	Female

Supplemental Material C: Codes[1]

Deductive Codes

D1: Binary and Rigid Thinking

D2: Doubts about Actions and Checking Behaviours

D3: Other-oriented Perfectionism

D4: Perfectionistic Self-presentation

D5: Self-Oriented Perfectionism

D6: Socially Prescribed Perfectionism

Inductive Codes

E1: Benefits of Perfectionism

E2: Challenges of Perfectionism

E3: Expectations, Standards and Goals

E4: Grades and Feedback

E5: Hard Work

E6: Need for Control and Organization

E7: Parents

E8: People Pleasing and Validation

E9: Prevalence

E10: Procrastination

E11: Social Comparison

E12: Social Interaction

E13: Strategies and Support

E14: Unattainable, Striving for More, Never Satisfied

[1] Please note that this is the full list of codes used for this study. Not all codes were used for the current analyses. Please see Table S4 and S5 in Supplementary Material D for which codes were analyzed in the current work.

Supplemental Material D: Audit Trail Tables

Table S4

Research Questions in Relation to Interview Questions, Interview Codes, and Open Response Questions

Research Question	Interview Questions	Codes	**Open-response questions**
1) What strategies do teachers employ when working with perfectionistic students?	P4	E13	"How do you deal with perfectionistic students? What strategies do you use?" [Q3]

Note. Codes beginning with 'E' represent inductive codes across interview transcripts
Note. See Supplemental Material B and C for a full list of interview questions and codes

Table S5

Themes in Relation to Questions and Codes

Themes	Questions/Codes
Growing Young Minds	P4, E13, Q3
It's About Time and Boundaries	P4, E13, Q3
Dismantling Perfectionism	P4, E13, Q3
Individualized Academic Support	P4, E13, Q3
Self-Care is Key	P4, E13, Q3

Note. Codes beginning with 'E' represent inductive codes
Note. See Supplemental Material A and B for a full list of interview questions and codes
Note. Each of these themes occurred in both question-based and thematic coding, and were therefore combined in analysis.

Supplemental Material E: Demographic Survey Questions Used for Determining Group Membership

Do you consider yourself to be a perfectionist? (Response options: Yes, No, Prefer not to say)

What is your gender? (Response options: Male, Female, Male-to-female Trans, Female-to-Male Trans, Non-binary/Gender Non-conforming, Other, Prefer not to say)

At which level do you teach? (Response options: Elementary, Secondary, Prefer not to say)

How many years have you been teaching? (Response options ranged from 1 year to 65+ years, in increments of 1 year, along with a Prefer not to say option)
